# Fertility preservation in gynaecologic cancers

**DOI:** 10.3332/ecancer.2018.798

**Published:** 2018-01-16

**Authors:** Francesca De Felice, Claudia Marchetti, Anna Di Pinto, Angela Musella, Innocenza Palaia, Maria Grazia Porpora, Ludovico Muzii, Vincenzo Tombolini, Pierluigi Benedetti Panici, Federica Tomao

**Affiliations:** 1 Department of Radiological, Oncological and Pathological Sciences, Sapienza University of Rome, Rome 00161, Italy; 2 Department of Gynecological and Obstetrical Sciences and Urological Sciences, Sapienza University of Rome, Rome 00161, Italy

**Keywords:** fertility preservation, gynaecologic cancers, ovarian cancer, cervical cancer, endometrial cancer

## Abstract

Due to substantial improvement in the diagnosis and treatment of gynaecologic cancers, a better understanding of patient care needs to be revised. We reviewed the literature related to fertility preservation strategies in gynaecological cancer and discussed current general management approaches. New technical modalities and patients’ own desire for motherhood should be integral and paramount in the clinical evaluation to significantly contribute to preserving fertility in those women diagnosed with gynaecologic cancers during the reproductive years.

## Introduction

Gynaecologic malignancies are relatively frequent in the female population, with a global estimated incidence of 222,700 new cases in Europe [1].

In the last decade, the percentage of patients who had a first pregnancy after 40 years was approximately 20% [2], resulting in a higher risk of cancer incidence before the satisfaction of the reproductive desire [3]. Thus, a considerable number of patients affected by gynaecologic tumours are of childbearing age at diagnosis and have not completed their wish to have a family.

However, compared to other types of tumours, the gonadotoxicity derived from medical and/or radiation treatment did not represent the main cause of infertility.

In fact, the diagnosis of a gynaecologic malignancy is able to affect the fertility capability from an anatomical point of view, to the extent that a definitive damage is caused by the surgical approach.

In this scenario, the consideration of fertility preservation represents an important issue [4–6].

For sure, treatment strategy must be optimised to guarantee tumour eradication and excellent clinical outcomes. However, patient’s survivorship wishes to preserve fertility should be taken into consideration and nowadays a personalised care plan should become paramount. Identification of patients with early stage disease, as well as new and improved therapeutic strategies, is key to the safe selection of patients for fertility-preserving treatment.

Fertility-sparing surgical strategies are feasible in early stage tumours, but advanced diseases usually require a radical approach leading to irreversible infertility, even if some authors hypothesised the futuristic use of uterus transplantation after an eventual previous oocyte cryopreservation [7–9].

Thus, most of the scientific evidence regarding fertility preservation in gynaecologic malignancies is mainly focused on surgical modulation.

In this context, we reviewed the literature related to onco-fertility in cervical, ovarian and endometrial cancers with particular attention to both radiation therapy (RT) and chemotheraphy (CHT) gonadotoxic effects. Moreover, we hereby discussed current general management approaches and related fertility preservation techniques, emphasising the need for multidisciplinary team involvement. Finally, we mentioned the emerging topic of fertility management of women with BRCA germline mutations and the psychological aspect of women with gynaecological cancer diagnosis. Indeed, despite many studies showing the connection between adequate counseling regarding fertility preservation treatments and the quality of life of women, there is no adequate information about this type of treatment [10].

## Radiation therapy

RT to the abdomen–pelvis is associated with a high risk of permanent amenorrhoea and can damage the uterus and uterine vessels, affecting the ability of a woman to carry a pregnancy to term [11].

The risk of radiation sequelae increases with an advanced stage disease, extensive residual tumour size and abdominal carcinomatosis. Improvement in radiation techniques, including intensity modulated RT (IMRT) and proton therapy, may allow the radiotherapist to spare the ovaries and uterus from significant radiation dose, minimizsing the potential adverse effects on fertility. Amenorrhoea and infertility can also be subsequent to cranial irradiation.

**Figure 1.**
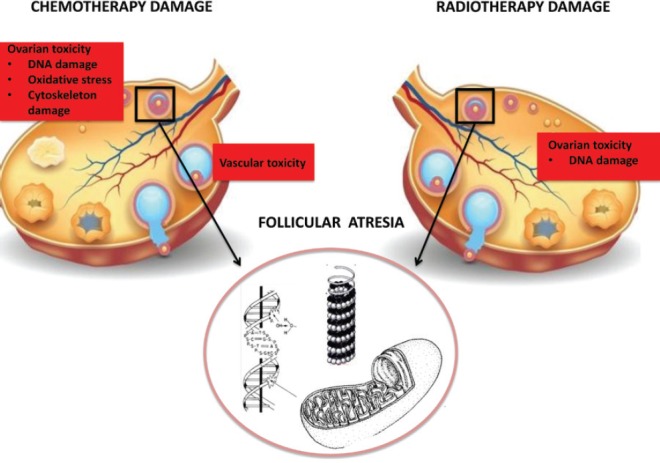
Ovarian damage.

*Ovarian dysfunction*: Abdomino-pelvic irradiation may directly affect the gonads, leading to infertility (Figure 1). The ovaries of a newborn girl contain approximately 2 million oocytes in the middle of a meiotic division, which becomes completed shortly before ovulation many years later [12]. The population of oocytes reduces with increasing age until menopause, when few oocytes survive.

Ionising radiation can determine direct DNA damage to ovarian follicles, resulting in follicular atrophy and thus decreasing ovarian follicular reserve. In general, oocytes are extremely radiosensitive. The effective sterilising dose, defined as the total dose at which premature ovarian failure occurs immediately after RT in 97.5% of patients, was found to decrease with increasing age at treatment, from 20.3 Gy at birth to 14.3 Gy at 30 years [13]. Radiation doses > 6 Gy in adult women older than 40 years of age are related to the highest level of risk for gonadal impact and immediate amenorrhoea. The individual variability in ovarian follicular reserves explains the differences in onset of ovarian failure after RT between patients treated at similar ages [14].

*Uterine dysfunction*: Uterine irradiation may predispose to pregnancy-related complications, including spontaneous abortion, preterm labour and delivery, malposition of the foetus, premature low birth weight and placental attachment disorders [15]. RT-related uterine dysfunctions are mainly due to myometrial fibrosis, damage to the uterine vasculature and endometrial injury. The prepubertal uterus is more sensitive than the adult uterus and its irradiation at a young age seems to reduce adult uterine volume, which may lead to adverse pregnancy outcomes [16]. Damage to the uterine vasculature may result in poor vascularisation and subsequently reduced feto-placental blood flow, which may impair fetal growth [14]. Placental abnormalities are related to endometrial injury and preclusion of normal decidualizsation.

*Hypothalamic–pituitary–ovarian axis dysfunction*: Hypothalamic–pituitary–ovarian axis dysfunction represents a well-known potential secondary effect of cranial irradiation. Its impact on the development of endocrinopathies depends on the RT-induced damage to the hypothalamus and/or the pituitary gland. By secreting gonadotropin-releasing hormone (GnRH), follicle-stimulating hormone (FSH), luteinising hormone (LH), estradiol, progesterone and prolactin, the hypothalamic–pituitary–ovarian axis is responsible for fine regulation of menstruation and fertility. However, this possible effect is of minimal concern to the vast majority of gynaecologic patients who do not present brain metastases at diagnosis. This issue is increasingly important in pediatric cancer survivors, but it is outside the scope of this review.

## Chemotherapy

The effect of chemotherapeutic drugs on ovarian function varies extensively according to age, as demonstrated for other tumours, and to CHT schedule (type, dose, number of cycles and mechanism of action of drug). The main common CHT agents used in gynaecological cancer are alkylating agents (cyclophosphamide and ifosfamide), platinum compounds (cisplatin and carboplatin), taxanes (paclitaxel), anthracyclines (doxorubicin) and antimetabolites (gemcitabine and 5-fluorouracil) [17]. Details are presented in Table 1.

Alkylating agents are gonadotoxic chemotherapeutic agents and have most consistently been associated with ovarian failure in a dose-dependent manner. The American Society of Clinical Oncology (ASCO) Clinical Practice Guideline Committee stated that women treated with high doses (≥5 g/m^2^) of alkylating agents have a high risk (more than 70%) of developing permanent amenorrhoea [4]. Alkylating agents determine oocytes damage via single-stranded DNA breaks and target cells at every stages of cell cycle, preferentially on primordial follicles [18]. The impact on fertility of taxanes and platinum is an intermediate risk level (30–70%) of amenorrhoea, whereas protocols containing antimetabolites and anthracyclines are related to lower risk (less than 30%) [4]. Additions of anthracycline to taxanes-based regimens appear to increase gonadotoxicity (Figure 1).

Most of the scientific evidence regarding the gonadotoxic effects of chemotherapeutic agents derives from clinical and experimental findings founded in other tumours.

Since the main cause of infertility in patients affected by neoplasia affecting gynaecologic organs is the surgical removal of the reproductive structures, few data are available about the effects of different chemotherapeutic agents in gynaecologic malignancies.

## Fertility preservation strategies

**Figure 2.**
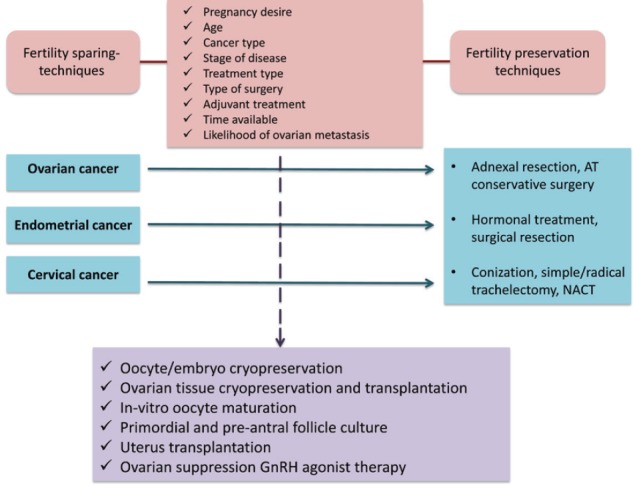
Fertility preservation techniques.

Fertility preservation strategies depend mainly on patient characteristics (age, desire to use male partner or banked donor sperm) and tumour characteristics (stage disease, histology, treatment). Furthermore, oncofertility counseling represents a crucial step to inform patients with cancer on the risk of developing treatment-induced premature ovarian failure and infertility with the proposed anticancer therapies and to present the different available options to preserve ovarian function and fertility by discussing the pros and cons of each option. A summary of the main fertility preservation techniques is provided in Table 2. In addition to the previous table, fertility preservation techniques for each gynaecological cancer are provided in Figure 2.

### Surgical and medical options

*Cervical cancer*: The appropriate strategy is based on each stage of the disease. Generally, conization and trachelectomy are performed in early cervical cancer (stage IA-IB1), whereas in the advanced stage (stage > IB1) the current standard recommendation is RT with or without CHT, although neoadjuvant CHT followed by radical surgery represents a valid alternative.

Regarding fertility preservation strategies, most of the international guidelines suggest that the first diagnostic and curative step for microscopic tumours consists of conization, a procedure consisting of the en bloc removal of the ectocervix and endocervical canals.

In the presence of negative margins and the absence of clinical contraindications to surgery, the cone biopsy may represent a definitive treatment.

Moreover, due to its extremely low incidence of lymphatic metastasis (<1%), conization alone without lymphadenectomy can be safely proposed in stage IA1 disease without the presence of lymphovascular space invasion (LVSI) [19].

In the case of LVSI, radical trachelectomy with laparoscopic pelvic lymphadenectomy is preferable, when fertility preservation is desired.

This technique includes surgical removal of the cervix to the level of the internal os, with en bloc resection of the vaginal fornix and supporting ligaments. The vagina is then sutured to the lower uterine body, and a cerclage suture is placed at the level of the internal os to prevent miscarriage during a future pregnancy [19]. In general, fertility sparing options in special histotypes are still being debated. After childbearing is complete, hysterectomy can be considered for patients who have had either conization or radical trachelectomy if human papilloma virus (HPV) infection persists [19].

Of note, some authors tested the use of conization eventually preceded by a neoadjuvant chemotherapy approach also in stage IA2, IB1 and even IB2 tumours with interesting results [20, 21].

In the case of primary definitive RT, ovarian transposition out of the radiation field is a treatment option to preserve ovarian function with a success rate of 70–100% [14]. At present, ovarian transposition, also known as oophoropexy, is a surgical laparoscopic procedure. The ovaries and fallopian tubes are detached from the uterus with their vascular pedicles intact and then placed in the iliac fossae as high and laterally as possible. The transposed ovaries may be sutured within the lateral paracolic gutter up the lowest rib, anterior to psoas muscles or in intra-abdominal paracolic gutters [22, 23]. This surgical procedure is linked to several complications, including Fallopian tube infarction, ovarian cyst formation and chronic ovarian pain. Moreover, it should be considered that regimens that include RT and CHT have an obvious negative impact on fertility. Therefore, candidate patients for ovarian transposition should be selected carefully.

*Ovarian cancer *: Most patients with ovarian cancer present with advanced disease that is not eligible for fertility preservation. However, borderline and early stage tumours, including stage I epithelial ovarian cancers, malignant ovarian germ cell tumours and sex cord stromal tumours, are potentially suitable for fertility-sparing surgical procedures. Since scientific evidence has showed that the rate of microscopic tumour in the contralateral ovary is very low, ranging from 0% to 2.5%, the contralateral ovary such as the uterus are retained. However, since the recurrence after conservative treatment, also in IA stage, is not an excluded hypothesis, some authors suggest the use of adjuvant platinum-based chemotherapy in order to limit this risk [3].

*Endometrial cancer*: The majority of endometrial cancer occurs in postmenopausal women. Endometrial cancer in women of reproductive age is often associated with a clinical history of polycystic ovarian syndrome – —which significantly reduces the chance of becoming pregnant and thus should be considered when proposing fertility-sparing treatment. In patients with stage IA disease who desire to preserve their fertility, a fertility-sparing treatment can be proposed [19]. Continuous progestin-based therapy followed by repeated endometrial curettage can be used in low-risk well differentiated endometrioid cancer with response rates varying from 42% to 100% and a recurrence rate around 24–40% [3]. Most of the studies about this topic suggested that treatment duration should be based on treatment response after at least 3 months; patients with persistent or progressive disease after 6–9 months should be considered for radical surgery [3]. Fertility preservation should not be recommended in the case of serous carcinoma, clear cell carcinoma or carcinosarcoma histology. However, if fertility preservation is chosen, hysterectomy is recommended after childbearing [19].

### Assisted reproductive options

Embryo and oocyte cryopreservation are the standard assisted fertility preservation strategies. To offer the opportunity to conserve fertility, cryopreservation should be performed before the start of cancer therapy.

*Embryo cryopreservation*: Embryo cryopreservation is the most established fertility preservation strategy if the patient has no urgency to start cancer therapy. A male partner or donor sperm is needed. Embryo cryopreservation is a safe technique. Both protocols, including randomly ovarian stimulation with subcutaneously injected gonadotropins for 8–14 days or within 3 days of the start of the menstrual cycle, are effective. After retrieval, oocytes are examined for maturity and then cryopreserved [24].

*Oocyte cryopreservation*: Unfertilised oocyte cryopreservation requires ovarian stimulation with daily gonadotropin injections and ultrasound-guided oocyte retrieval under sedation. This technique is particularly important for patients who do not have a male partner or prefer not to use donor sperm [4]. Compared to embryo cryopreservation, oocyte freezing is still associated with lower pregnancy rates (4.6–12% versus 30–40%) [17, 24].

*Ovarian cryopreservation*: Ovarian tissue cryopreservation is currently considered an experimental technique to preserve reproductive potential [25]. Ovarian cortex is rich in quiescent primordial follicles with oocytes arrested in the diplotene of prophase of the first meiotic division. Good candidates for this procedure are women younger than 40, because of higher ovarian reserve. No partner is needed and no ovarian stimulation is required, thus there is no delay in the treatment onset [24]. Technically, by a laparoscopic approach, cortical ovarian tissue from 0.5 to 2 cm is obtained and then cryopreserved for either later reimplantation or *in vitro* maturation of eggs [4].

### Other strategies

*Gonadotropin-releasing hormone agonists*: The use of gonadotropin-releasing hormone agonists (GnRHa) is an investigational strategy, based on the use of GnRHa to suppress ovarian function during CHT. Theoretically, the use of GnRHa reduces the gonadotropins level, placing the ovaries in an artificial prepubertal state [26]. Moreover, studies of ovarian tissue have led to the hypothesis that these compounds could be able to reduce the organ damage and the local ischaemia caused by chemotherapeutic agents. Ideally, its administration should be started 2–4 weeks before CHT. Nonetheless, the solid rational regarding the application of these agents for the preservation of ovarian failure caused by chemotherapy, their role, as well as the efficacy are still debated, with conflicting results [27]. However, the most recent evidence seems to demonstrate the protective role of GnRHa against early menopause onset with a favorable pregnancy rate [28]. However, it must be considered that GnRHa are related to menopausal-like symptoms, including hot flushes, headaches, mood changes, sweating and decreased bone density.

Their application has been largely investigated in fertility preservation of patients with breast cancer, but few data are available about their use in gynaecologic malignancies.

However, a potential role for fertility preservation of gynaecologic malignancies might be hypothesised for the preservation of ovarian failure after a conservative surgical treatment of ovarian cancer or for the use of neoadjuvant approaches before a conservative surgical procedure for early stage cervical tumours.

*Actual and future perspectives for hysterectomised women (beyond the uterus transplantation)*:All the strategies reported until now may be considered in early stage tumours not requiring the removal of reproductive organs. The only motherhood options for women who undergo hysterectomy are adoption (to acquire legal motherhood) or pregnancy in a gestational surrogate carrier after oocyte or embryo preservation, if applicable, in order to acquire genetic motherhood, followed by adoption to achieve legal motherhood.

Despite the interesting results reported by Brännström *et al* [8] about the use of uterus transplantation in either benign and malignant conditions, the use of high doses of immunosuppressive agents, the risk of cancer recurrence in immunocompromised patients, and the possible vascular abnormalities after pelvic radiation must be reviewed before taking this approach into consideration in cancer patients.

However, it could represent a revolutionary approach for the management of gynaecologic cancer patients with fertility preservation purposes.

## Fertility issues in BRCA carriers

It is well established that women with germline mutation BRCA genes have an increased lifetime risk of developing ovarian and breast cancer. To minimise cancer risk and maximise survival, risk-reduction surgeries should be performed at 25 years old [29]. Although women with BRCA mutations are more likely to develop breast cancer at a younger age than ovarian cancer, the decision to opt for bilateral risk-reducing mastectomy (BRRM) and risk-reducing salpingo-oophorectomy (RRSO) should be performed according to evidenced-based knowledge of the risks and benefits of prophylactic surgical procedures [30]. For sure, RRSO is recommended to prevent ovarian cancer/fallopian tube cancer in BRCA mutation carriers by 40 years of age or after completion of childbearing [31]. This preventive strategy implies significant limitation in reproductive potential and may impose health risks due to early menopause [32].

In addition, controversy exists about fertility potential in this setting of patients. In fact, BRCA germline mutations seem to be related to a lower ovarian reserve – and thus a shorter reproductive lifespan – compared to women without BRCA mutations. The loss of BRCA function predisposes to DNA damage and subsequent accelerated follicular atresia and apoptotic oocytes [33]. At present, early fertility counseling is recommended.

## Psychological impact of gynaecological cancer

Cancer is one of the diseases most commonly accompanied by psychosocial problems, and cancer patients may suffer, over the long term, from psychosocial disorders like fatigue, sleep disorders, cognitive and functional limitations, sexual dysfunctions, infertility, psychological problems, and psychiatric disorders [34, 35]. In gynaecological cancers, women can experience suffering with negative effects in the emotional sphere, thanks to the association with cancer diagnosis to death [36]. In addition, because of the treatments that are often radical based on the stage, with a physical and hormonal upheaval, they develop problems associated with sexual dysfunction, body image disorders, decreased quality of life, and psychological diseases such as anxiety and depression [37]. One study showed that 21.3% of the women diagnosed with cervical cancer suffered from depression, and that 6% of the women diagnosed with ovarian cancer had depressive symptoms [38].

Depression may have a negative impact on patients’ daily life and communication with their environment. McCorkle *et al* [39] showed in 2006 that women with gynaecological cancer who have changes in their marriages, work or financial statuses have a greater tendency towards depression. An important deterioration in the quality of life contributes to the onset of depression in these patients. In fact, many studies on this topic have demonstrated that the consequences of cancer and its treatment may be associated with a diminished level of QoL and that low levels of QoL are correlated with higher levels of anxiety and depression [40].

It’s important to identify patients with particular risk of developing sexual and psychological problems. It would always be advisable to carry out an interview, using the Brief Index of Sexual Functioning for Women [41] or the Female Sexual Function Index (FSFI) [42], about sexual function before cancer, current sexual activity, and how cancer modifies sexual health and relationship with the partner [43].

## Conclusion

The real effectiveness of different strategies for fertility preservation of gynaecologic malignancies needs to be determined. Since the main cause of infertility consists of surgical removal of the reproductive organs, a modulation of the surgical approach is the most desirable procedure.

Embryo and oocyte cryopreservation are considered standard practice and are widely available in most other tumours; these procedures can also be used in gynaecologic cancers, for example in cases of uterus sparing or in cases of uterus-surrogate carriers. Other fertility preservation strategies should be considered experimental and should be reserved for patients who are treated in specialised centres with the necessary expertise.

Although patients and physicians should be focused initially on cancer diagnosis, the patient and her family should be extensively counseled for fertility preservation. The risk of conservative therapy balanced against the disadvantages of more radical treatment should be explained. The patient should realize that by choosing fertility-sparing treatment, she is assuming a less but not well-defined risk of recurrent disease. Moreover, patients and physicians should evaluate the realistic probabilities of achieving conception based on the patient’s age, as well as her family history and fertility evaluation.

A multidisciplinary team (gynaecologic oncologists, radiation oncologists, medical oncologists, reproductive endocrinologist and perinatologist) evaluation should be the standard. Therefore, the presence of a psychologist is important to convey the correct information to patients with gynaecological cancer who wish to preserve their reproductive capacity [44, 45].

## Figures and Tables

**Table 1.** Chemotherapy agents used in gynaecological cancer.

Drugs		Mechanism of action	Target cell	Risk of amenorrhoea
**Alkylating agents**		Single-stranded DNA breaks	Non primordial follicles Primordial follicles Follicular atresia	High
	Cyclophosphamide			
	Ifosfamide			
**Platinums**		Inter- and intra-strand DNA cross-links; chromosomal damage	Oocyte	Intermediate
	Cisplatin			
	Carboplatin			
**Taxanes**		Inhibition of microtubule formation and spindle functions	Primordial follicles Granulosa cells	Intermediate
	Paclitaxel			
**Anthracyclines**		Inhibition of DNA repliction and trascription	Stromal tissue Granulosa cells	Low
	Doxorubicin			
**Antimetabolites**		Alteration DNA synthesis	Pre-antral Antral	Low
	Gemcitabine			
	5-fluorouracil			

**Table 2.** Fertility preservation strategies in gynaecological cancer.

Strategy		Definition
**Surgical option**		
	Conization	Removal of the ectocervix and endocervical canals
	Radical trachelectomy	Removal of the cervix with preservation of the uterus
	Ovarian transposition	Transposition of ovaries out of the radiation field
	Unilateral oophorectomy	Removal of one ovary
**Medical option**		
	Progestin-based therapy	Progestin therapy and endometrial curettage
	GnRHa	GnRHa-based therapy
**Assisted option**		
	Embryo cryopreservation	In vitro fertilisation of oocytes, then cryopreserved
	Oocyte cryopreservation	Unfertilised oocyte cryopreservation
	Ovarian cryopreservation	Removal of ovarian tissue, then cryopreserved
